# METHODOLOGICAL AND ETHICAL ISSUES OF RESEARCH WITH OLDER ADULTS IN PUERTO RICO DURING COVID-19

**DOI:** 10.1093/geroni/igac059.620

**Published:** 2022-12-20

**Authors:** Sunghwan Cho, David Camacho, Thomas Buckley, Julia Vazquez, Kiara Carrasquillo-Sánchez

**Affiliations:** Virginia Commonwealth University, Richmond, Virginia, United States; University of Maryland, Baltimore, Norwalk, California, United States; University of Pittsburgh, Pittsburgh, Pennsylvania, United States; University of Maryland, Baltimore, Baltimore, Maryland, United States; Seneca Family of Agencies, Oakland, California, United States

## Abstract

Engaging diverse groups of older adults is essential to addressing health disparities. In this presentation, we will describe how we used our research team’s networks and knowledge of the target population to engage key stakeholders in our KAP project. We will address our partnerships with clergy, NGO staff and administrators, housing authorities, colleagues at Puerto Rican universities, and older adults who serve as informal community and institutional gatekeepers. We will discuss how these partnerships facilitated our ability to identify and access study sites and participants; conduct telephone and in-person interviews; ensure data quality through training and monitoring; and, assure safety through adherence to COVID-19 protocols. Finally, we will describe key cultural and ethical issues of conducting research with older adults through community partnerships during a pandemic. The presentation has implications for developing beneficial partnerships with local community leaders and enhancing the representation of diverse groups of older adults in research.

